# Recurrent Intestinal Obstruction after Radiation Therapy: A Case Report and Review of the Literature

**DOI:** 10.1155/2019/5198958

**Published:** 2019-03-06

**Authors:** James Nguyen, Amani Jambhekar, Ziyad Nasrawi, Prasad Gudavalli

**Affiliations:** Department of Surgery, New York Presbyterian Brooklyn Methodist Hospital, Brooklyn, New York, USA

## Abstract

**Introduction:**

In patients who have undergone resection for rectal cancer after neoadjuvant radiotherapy, loop ileostomy is commonly performed with few serious complications. In rare cases, if this irradiated small bowel is strictured, reversal of the affected ileostomy can have dire consequences. We present a case of a 62-year-old male with recurrent intestinal obstruction after closure of his loop ileostomy.

**Case Report:**

RC is a 62-year-old male who initially presented with rectal cancer and underwent neoadjuvant chemoradiation prior to a laparoscopic low anterior resection with diverting loop ileostomy. He underwent elective reversal of his ileostomy and developed persistent postoperative obstruction. He underwent resection of the prior reversal site with normal-appearing dilated proximal bowel loops and collapsed distal bowel loops. He again developed an obstructive picture and underwent resection of the prior anastomosis with creation of an ileocolic anastomosis, after which he recovered well postoperatively.

**Conclusion:**

In patients who receive radiation adjuvant therapy for colon cancer, radiation-induced stricture should be considered as a cause of small bowel obstruction postoperative. In the setting of a longstanding ileostomy, evaluation of a defunctionalized distal ileum may be necessary to evaluate potential obstruction from radiation changes.

## 1. Introduction

Radiation enteropathy is a functional disorder of the intestine that occurs during or after a course of radiotherapy to the abdomen, pelvis, and rectum [1]. It is etiologically complex and not solely attributable to overdosage or technique and may manifest at any time point postoperative in cases of concurrent surgery [2]. In patients who have undergone resection for rectal cancer with neoadjuvant radiotherapy, loop ileostomy is commonly performed with few serious complications despite being involved in the irradiated field [3, 4]. Stricture formation is recognized as one of the common complications of chronic radiation enteritis [5]. If this irradiated small bowel is strictured and bowel continuity is restored by closing the loop ileostomy, the consequences can be dire resulting in need for further surgical treatment [4]. We present a case of a 62-year-old male with recurrent intestinal obstruction after closure of his loop ileostomy.

## 2. Case Report

RC is a 62-year-old male who initially presented with a T3N2M0 midrectal cancer and underwent neoadjuvant chemoradiation four weeks prior to a laparoscopic low anterior resection with diverting loop ileostomy. He completed adjuvant chemotherapy and returned for an elective reversal of his ileostomy nine months postoperative. His preoperative workup included a colonoscopy which revealed exclusion colitis for which he was treated. He also underwent a gastrografin enema and computed tomography (CT) imaging of his abdomen and pelvis with no evidence of recurrence, obstruction, or distant metastases. On CT imaging, the proximal ileum appeared normal, but the distal ileum was not imaged. After his reversal, his postoperative course was complicated by persistent small bowel obstruction, for which he was managed conservatively for two weeks. He subsequently underwent a CT abdomen and pelvis, which was highly suspicious for anastomotic stricture.

**Figure 1:**
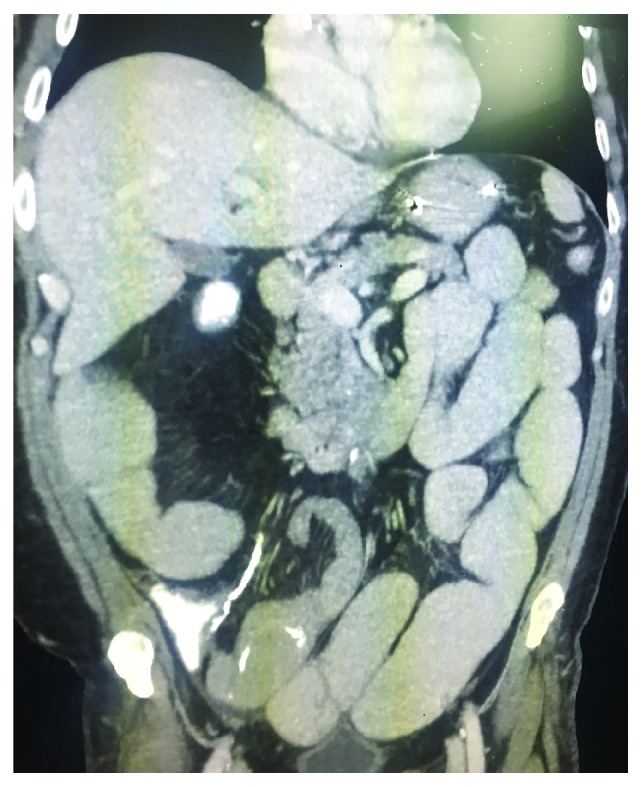
CT imaging showing narrowing of the ileum distal to the previous anastomosis.

**Figure 2:**
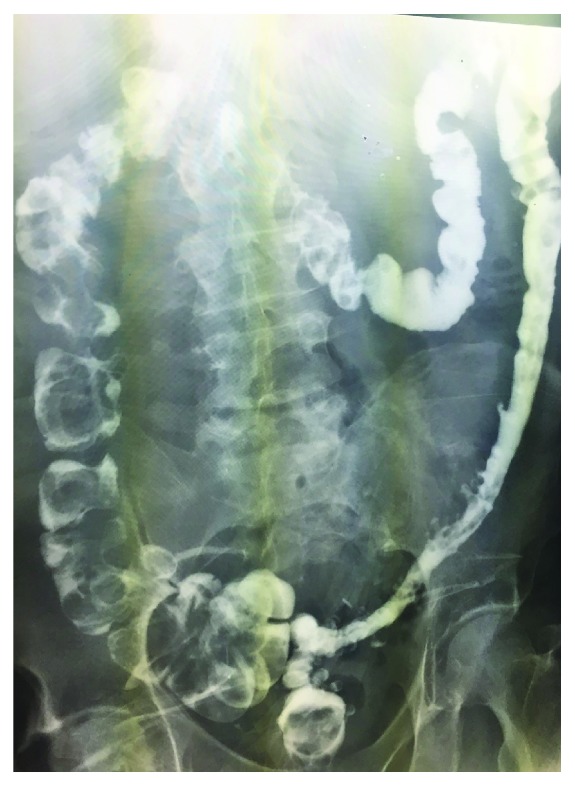
Small bowel series showing a narrowing of the ileum distal to the previous anastomosis.

**Figure 3:**
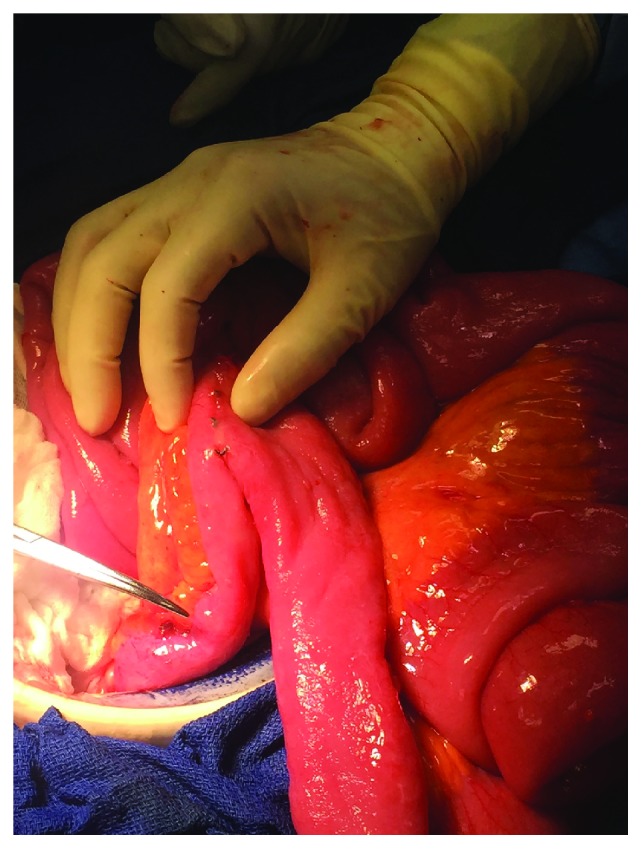
Intraoperative image of the thickened distal ileum.

**Figure 4:**
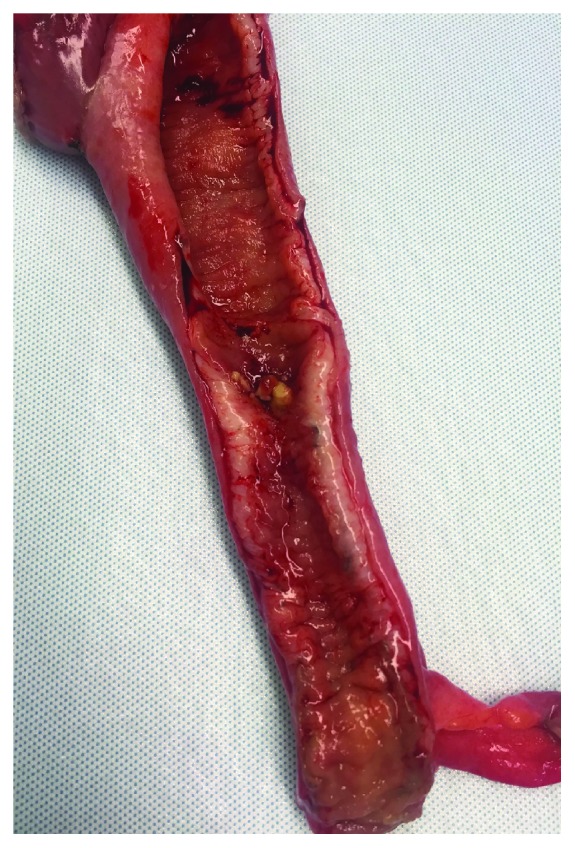
Small bowel specimen with stricture.

On postoperative day 14, the patient underwent a diagnostic laparoscopy showing multiple adhesions around the previous reversal site with normal-appearing dilated proximal and collapsed distal small bowel. There was no localized stricture in the defunctionalized distal ileum. He underwent a resection of the prior reversal site and creation of a new side-to-side primary anastomosis. Despite creation of new anastomosis, his small bowel obstruction continued for additional two weeks. CT imaging and small bowel series were obtained, both modalities showing a narrowing of the ileum distal to the previous anastomosis (Figures 1 and 2). On hospital day 27, the patient underwent a final exploratory laparotomy with intraoperative findings of persistent collapsed bowel loops distal to the new anastomosis. The collapsed distal segment was resected, and an ileocolic anastomosis was created. On gross examination, the entire distal ileum was thickened without stricture (Figures 3 and 4). The pathology of the distal ileum showed submucosal fibrosis with hyalinization of the lamina propria and atherosclerotic changes in the adjacent vessels. After the second revision, the patient progressed as expected with return of bowel function and tolerance of diet and was later discharged on hospital day 38. Our presented patient continues to do well on the outpatient follow-up.

## 3. Discussion

Radiation-induced small bowel injury has been reported with an incidence of 0.8-17%, but true incidence is difficult to determine with underrepresentation in the literature [2, 4]. Radiation-induced bowel injury typically presents with bleeding, diarrhea, fistula formation, and rarely, perforation [5]. Several prior reports have been published regarding patients suffering complication status postadjuvant radiation therapy after surgical resection for colon cancer. Morris and Haboubi describe pelvic radiation disease (PRD) as increasing the risk of bowel wall stricture formation, adhesions, fissures, and severe bleeding and bowel wall perforation [6]. Zakaria et al. report two patients who developed small bowel obstruction from radiation-induced strictures after reversal of a diverting ileostomy; both patients were treated with resection of the affected area and reanastomosis [4]. Our patient underwent preoperative imaging to evaluate the patency of the distal bowel prior to reversal of ileostomy which failed to include the distal ileum. Upon reversal, he developed an obstructed picture and after two consecutive operations was found to have a stricture of the distal ileum on pathology. This was not evidence on initial diagnostic laparoscopy as the bowel appeared grossly normal. Multiple adhesions were present with tortuosity of the small bowel around the ileostomy reversal, initially thought to be the point of obstruction. After the patient did not progress, postoperative imaging showed narrowing of the bowel, which led to repeat operation and resection of the affected segment. Upon back table examination, the intraluminal thickening was clearly palpable, though the bowel itself appeared healthy without evidence of ischemia or localized stricture. Pathologic evaluation confirmed narrowing secondary to radiation-induced ischemia. The surrounding adhesions and grossly normal appearance of the affected segment led to the delay in diagnosis. Our patient shows that in the context of abdominopelvic radiation, previously undiagnosed long segment strictures secondary to pelvic radiation disease can lead to small bowel obstruction which may be missed without complete radiological evaluation. This case has exemplified the need for complete imaging in this patient population prior to reversal of ileostomy including gastrografin enema and CT imaging with oral and intravenous contrasts. Furthermore, one may consider the increased specificity of evaluating the distal limb of the ileostomy with gastrografin contrast to identify nonfunctionalized small bowel secondary to pelvic radiation disease.

## 4. Conclusion

In patients who receive radiation adjuvant therapy for colon cancer, radiation-induced changes should be considered as a cause of small bowel obstruction postoperative. Complete radiologic workup before diverting ileostomy reversal is necessary to evaluate distal bowel patency for reanastomosis but may miss potential radiation-induced pathology. In the setting of a longstanding ileostomy, evaluation of the patency of the defunctionalized distal ileum may be necessary to evaluate potential obstruction from radiation changes. These patients can be successfully treated with resection of the affected segment and reanastomosis.
